# A Comparative Study on the Prevalence of Lifestyle Risk Factors Among Users and Non-users of Any Health-Based Mobile App Among University Students in Chennai

**DOI:** 10.7759/cureus.56203

**Published:** 2024-03-15

**Authors:** Rushender Rajan, Logaraj Muthunarayanan

**Affiliations:** 1 Preventive and Social Medicine, St. Peter's Medical College, Hospital and Research Institute, Hosur, IND; 2 Community Medicine, SRM Medical College Hospital and Research Centre, SRM Institute of Science and Technology, Kattankulathur, IND

**Keywords:** hypertension, healthy diet, who-steps, adolescents, mobile health apps

## Abstract

Introduction

Adolescents and young adults are often neglected in terms of healthcare, despite forming a significant demographic and economic force. This study aims to compare the prevalence of lifestyle risk factors among users and non-users of health-based mobile apps among university students in Chennai.

Methods

This cross-sectional study was conducted from January 2019 to June 2019, with a sample size of 680 undergraduate college students. A standardized WHO STEPwise approach to noncommunicable disease (NCD) risk factor surveillance (WHO STEPS) questionnaire was used to collect data on sociodemographic features, behavioral risk factors, and mobile app usage, along with physical measurements like height, weight, waist circumference, and blood pressure.

Results

About 238 (35%) study participants were regularly using health-based mobile apps, and 442 (65%) were not using any health-based mobile apps, of which 540 (79.41%) were between 22 and 24 years old, with the majority being female (362, 53.2%). The prevalence of various risk factors was higher among non-users of mobile health apps, except for physical activity, which was similar in both groups. On adjusted multivariable analysis, age category, place of current stay, and unhealthy diet were found to be significant.

Conclusion

This study helps assess the efficacy of health-based mobile apps in promoting healthy lifestyles among university students. Health-based mobile apps for delivering effective healthcare services are to be promoted among college students for a healthy lifestyle and well-being.

## Introduction

Adolescents are young people aged between 10 and 19 years, and people aged between 20 and 24 years are considered young adults. Globally, the population of adolescents is about 1.2 billion, and about 21% of the Indian population is made up of adolescents (about 243 million). Together with young adults, they contribute to more than one-third of the Indian population. They are the future of any nation, forming a major demographic and economic force [1].

Even though they are a major portion of the population, the health of adolescents and young adults is one of the least focused aspects. The general assumption that this group is at the prime of their lives and hence not prone to ill health is one of the major reasons for this. The lack of adequate studies and the resulting poor understanding of their health problems is also another major reason for this neglect [2-4]. Young adults in university education, however, frequently experience several difficulties, including adjusting to a new environment, stress from their academics, poor time management, and busy class schedules that lead them to be ignorant of their health [5]. Many studies across the globe have highlighted the onset of various high-risk behaviors, which increase the risk of chronic diseases and premature death during this period [6-9]. The most important of these behaviors include insufficient physical activity, unhealthy dietary practices, and substance abuse, including tobacco and alcohol use, which are well-recognized risk factors for future non-communicable diseases, including coronary artery diseases, stroke, cancer, etc. [10-12]. These risk factors are much higher in adolescents and young adults belonging to higher socio-economic strata of society, living in urban and metropolitan areas, compared to their rural counterparts [13]. Many Western countries give top priority to adolescent health problems. But in India, so far the main focus of adolescent health has been on reproductive health [14,15]. The pattern of high-risk behaviors that increase the risk of future chronic diseases is poorly understood due to the scarcity of studies on the subject.

Massive advancements in technology are one of the chief contributing factors to the increasing untoward changes in lifestyle. Many recent systematic reviews have been published in recent times evaluating the types of health (mobile health) interventions or applications, the diseases being targeted by them, and their efficacy. The overall evidence from these reviews shows that mobile health (mHealth) interventions are effective in achieving the desired behavioral change, even though to a variable extent. Especially lifestyle-related factors like diet, physical activity, etc. are reported to be effectively addressed by these interventions [16-18]. However, the key limitation of the majority of the mHealth interventions was the predominant “one-way” nature of communication. Participant involvement is more passive and is often limited to following the instructions or the tips given by the application [16].

Hence, there is a vital need to combine evidence-based interventions, which have been proven to be very effective in achieving and sustaining behavioral change, advanced technologies, and medical knowledge to evolve useful interval interventions. This will facilitate the active involvement of the participants in setting up their own behavioral goals and an implementation plan. This may help in grossly enhancing the efficacy of the mHealth interventions. Thus, we have done a comparative study among mHealth app users and non-user adolescents to estimate the prevalence of lifestyle risk factors and assess the effects of the health-based mobile app among them.

## Materials and methods

A cross-sectional study to estimate the noncommunicable disease (NCD) risk factors among university students was conducted from January 2019 to June 2019 in the Chengelpattu district of Tamil Nadu. This study is part of a large-scale pre-post study. The study was conducted at SRM Medical College and Hospital, SRM Institute of Science and Technology, Chengalpattu, Chennai. The study population included undergraduate college students who were willing to participate in the study. The sample size was calculated based on the previous study conducted by Chakma et al. [19]. The proportion of college students with high-risk behavior is 28.66%, relative precision is 7.5%, the design effect is 1.5, and the non-response rate is 10%. The required sample size was 311, approximately equal to 320.

Also, to evaluate the intervention, using pre-post intervention to detect 5% change after the intervention, with 80% power and a 2-sided alpha error of 5%, a correlation of 50%, the required sample size would be 678. The sample size was calculated using STATA IC, Version 14, Statistical Software, College Station, TX: StataCorp LP. A simple random sampling technique was used to select the required number of students. The selected study participants were organized based on the course of study and the year of the study. A standardized and validated WHO STEPwise approach to NCD risk factor surveillance (WHO STEPS) questionnaire was used for data collection [20]. It consists of the following parameters: Step 1 deals with information on sociodemographic features and behavioral risk factors (tobacco use, alcohol consumption, physical inactivity, and fruit and vegetable intake), as well as the history of NCDs and related conditions such as raised blood pressure, diabetes, raised cholesterol, cardiovascular diseases, etc., which is collected through self-report. Step 2 gathers data through simple physical measurements like height, weight, waist circumference, and blood pressure. A structured questionnaire (mobile application utilization questionnaire) was used to collect data regarding mobile phone ownership, type of mobile, presence of internet connectivity, hours and patterns of mobile phone usage, health-based app usage, etc. For the students’s convenience, they were offered three different timeslots, one each in the morning, afternoon, and evening hours, to collect the data.

The study was approved by the Institutional Ethics Committee of SRM Medical College (approval number: 1665/IEC/2019). Students were explained about the purpose and methodology of the study. A written informed consent from each student was obtained before administering the questionnaire.

Statistical analyses

All data was entered in Microsoft Excel (Microsoft Corporation, Redmond, Washington, United States), and analysis was carried out using IBM SPSS Statistics for Windows, Version 26 (released 2019; IBM Corp., Armonk, New York, United States). Descriptive analysis was carried out by frequency and proportion for categorical variables. Continuous variables were presented as mean ± SD for normally distributed variables and median (IQR) for non-normal variables. The chi-square test was used to test the statistical significance of cross-tabulations between categorical variables.

Using log-binomial regression, the relationship between the health-based mobile app users and each of the independent variables from the WHO STEPS questionnaire was evaluated. The results were presented as a prevalence ratio (PR) with a 95% confidence interval (CI). For multivariate logistic regression, only the variables that showed statistical significance at a p-value <0.2 in log-binomial regression were taken into account. P-values <0.05 were considered statistically significant in the adjusted analysis.

## Results

The total number of participants recruited for the study was 680. Table 1 describes the mobile application and usability data among the study participants, which shows that 559 (82.2%) were using Android mobile, with the majority (573, 84.2%) using mobile data. About 238 (35%) of study participants were regularly using health-based mobile apps, and 442 (65%) were not using health-based mobile apps.

**Table 1:** Mobile application and usability data among the study participants (N=680)

Parameter	N(%)
Which category of smart mobile device do you own?	
iPhone Mobile	121(17.79)
Android Mobile	559(82.21)
Which of the following internet access do you utilize for your device more often?	
Mobile data	573(84.26)
Wi-fi	107(15.74)
Do you search for health-related news/articles and videos on your mobile phone?	
No	129(18.97)
Yes	551(81.03)
Are you aware of health-related apps?	
No	169(24.85)
Yes	511(75.15)
If yes, have you installed any of them on your mobile phone?	
No	312(45.88)
Yes	368(54.12)
If installed, are you utilizing them regularly?	
No	442(65.00)
Yes	238(35.00)
How long have they been using the app regularly? N=238	
Less than or equal to one year	68(28.5)
More than one year	170(71.5)

Overall, the age groups of the population that participated in the study were 22-24 years old (540, 79.41%) and 25-29 years old (140, 20.59%), respectively. Female participants were slightly higher in number (362, 53.24%) compared to male participants (318, 46.76%). Around 475 (69.85%) are day scholars, and 205 (30.15%) of study participants are from hostels. It is evident that 55 (8.09%) participants use any form of tobacco product (cigarettes, cigars, or pipes) and 6 (0.88%) use smokeless tobacco products (snuff, chewing tobacco, and betel). About 179 (26.32%) study participants have consumed alcoholic drinks in any part of their lifetime, of which 109 (16.03%) have consumed within the past 30 days. Table 2 shows the same classification based on health-based mobile app users and non-users, and most variables are similar when compared between the groups given with their p-values >0.05. 

**Table 2:** Risk factor profile of the study participants among those regularly utilizing health based mobile apps (N=680) *Asian-Pacific Classification of body mass index (BMI)

Parameter	Non-user frequency (%) (n=442)	User frequency (%) (n=238)	p-value
Age groups			
22-24 years	328(74.2)	212(89.1)	0.02
25-29 years	114(25.8)	26(10.9)	
Gender			
Female	224(50.6)	138(57.9)	0.07
Male	218(49.4)	100(42.1)	
Place of current stay			
Day scholar	298(67.4)	177(74.3)	0.06
Hostel	144 (32.6)	61(25.7)	
Tobacco usage in last 30 days			
Yes	0(0)	61(13.8)	<0.01
No	238(100)	381(86.2)	
Alcohol consumption in last 30days			
Yes	18(7.6)	91(20.6)	0.04
No	220(92.4 %)	351(79.4)	
Unhealthy diet			
Yes	74(31.1)	266(60.2)	0.02
No	164(68.9)	176(39.8)	
Physical inactivity			
Yes	185(77.7)	341(77.15)	0.8
No	53(22.3)	101(22.85)	
BMI Category*			
Severely underweight	0(0)	9(2.1)	0.6
Underweight	1(0.4)	26(5.9)	
Normal	133(55.8)	237(53.6)	
Overweight	91(38.2)	140(31.6)	
Obese	13(5.6)	30(6.8)	
Hypertension status			
Yes	15(6.3)	44(9.9)	0.1
No	223(93.7)	(90.1)	

Table 3 shows the prevalence of various risk factor profiles, including tobacco, alcohol consumption, unhealthy diets, physical activity, obesity, and hypertension, among university students as a proportion with a 95% confidence interval, which clearly shows that the prevalence was higher in non-users than any health-based mobile app users, with none having any form of tobacco consumption, while physical inactivity remained similar in both groups. 

**Table 3:** Prevalence of risk factor parameters in the study participants among the groups (N=680) *Asian-Pacific Classification of body mass index (BMI); CI: confidence interval

Parameters	Non-users prevalence (95%CI)	Users prevalence (95%CI)
Hypertensive	9.9(7.4 to 13.1)	6.3(3.7 to 9.9)
Obesity*	6.8(4.7 to 9.4)	5.6(3.1 to 8.9)
Tobacco use	13.8(10.8 to 17.1)	0(0)
Alcohol use	20.6(17.1 to 24.5)	7.6(4.7 to 11.4)
Unhealthy diet	60.2(55.6 to 64.6)	31.1(25.4 to 37.1)
Physical inactivity	77.1(73.1 to 80.1)	77.7(72.1 to 82.6)

On unadjusted analysis with log-binomial regression, age category, gender, place of current stay, tobacco usage, alcohol consumption, unhealthy diet, and hypertension status were found to be significant with p-values <0.2. On further adjusted multivariable analysis, participants in the age group 22-24 years had a 45% higher proportion (adjusted PR = 1.45, 95% CI = 1.31 to 1.65) of using health-based mobile apps than those aged 25-29 years, and likewise, those without alcohol consumption, hostelers, and participants with healthy diets had an increased proportion of using health-based mobile apps and were statistically associated factors with a p-value of <0.05 as shown in Table 4.

**Table 4:** Regression analysis of sociodemographic and risk factors among university students using health-based mobile apps (N=680) *Asian-Pacific Classification of body mass index (BMI); CI: confidence interval #Systolic BP>140mmHG; Diastolic BP>90mmHG

Socio-demographic and behavioral characteristics	Total, N	Mobile health app users, N(%)	Unadjusted prevalence ratio	95% CI	p-value	Adjusted prevalence ratio	95% CI	p-value
Age category								
22-24 years	540	212(39.2)	1.47	1.32-1.67	<0.001	1.45	1.31-1.65	<0.001
25-29 years	140	26(18.5)	Ref	Ref		Ref	Ref	
Gender								
Male	318	100(31.4)	Ref	Ref	-	Ref	Ref	0.33
Female	362	138(38.1)	1.34	0.97-1.84	0.06	0.89	0.71-1.12	
Place of current stay								
Day scholar	475	177(37.2)	0.71	0.51-1.01	0.06	0.73	0.56-0.95	0.02
Hostel	205	61(28.9)	Ref	Ref	-	Ref	Ref	
Alcohol consumption								
Yes	109	18(16.5)	Ref	Ref	-	Ref	Ref	<0.001
No	571	220(38.5)	0.31	0.18-0.53	<0.001	0.38	0.24-0.60	
Unhealthy diet								
Yes	430	74(29.6)	Ref	Ref	-	Ref	Ref	0.04
No	250	164(38.1)	1.46	1.04- 2.04	0.02	1.23	1.01- 1.72	
Physical Inactivity								
Yes	526	185(24.1)	1.02	0.72-1.34	0.87	-	-	-
No	154	53(23.8)	Ref	Ref	-	-	-	
BMI category*								
Non-obese	637	225(35.3)	0.85	0.56 – 1.34	0.53	-	-	-
Obese	43	13(30.2)	Ref	Ref	-	-	-	
Hypertension status#								
Yes	59	15(25.2)	Ref	Ref	-	Ref	Ref	0.32
No	621	223(35.7)	0.71	0.46 – 1.14	0.12	0.79	0.61 – 1.34	

## Discussion

Unhealthy lifestyle behaviors, such as poor diet, lack of physical activity, smoking, and alcohol consumption, are prevalent among university students and can have negative impacts on their overall health and well-being. Various studies have shown that university students are at risk of developing NCDs at a very young age [21,22].

The current study found the prevalence of current tobacco use (smoke and chewable tobacco) at 8.97% among the study population, which was quite higher when compared to the Global Adult Tobacco Survey (GATS-2) survey (2016-2017) when the prevalence of use of any form of tobacco in those aged 15-24 years was 2% [23]. Similar findings were reported among university students in a study conducted by Jai Soorya et al. that used the alcohol, smoking and substance involvement screening test (ASSIST) for assessing tobacco use, and its severity was reported at 8.6%, a study by Menon et al [24]. The prevalence of alcohol consumption in the present study is found to be 16.03 percent, which is in line with the study conducted among undergraduate medical students, where 16.7% of students are alcohol users [21]. Obesity is an important NCD risk factor that is linked to an increased risk of hypertension, stroke, coronary heart disease, and cancer. Our study found that the prevalence of obesity was 16.6% among the study population. A similar study conducted among college students in Iraq found a lesser prevalence of 10.67%, respectively [25]. In this study, the prevalence of hypertension is 4%, which is comparatively less than other NCD risk factors. This is in contrast with studies conducted in other parts of India, where the prevalence was found to be 26.2% (Haryana) [26], 15.6% (Uttar Pradesh) [27], and 17.8% (West Bengal) [28]. The change may be due to different dietary habits and lifestyle modifications.

The usage of health-based mobile apps was significantly higher among younger participants. Bol et al. observed almost comparable results, showing that most users were either younger (odds ratio (OR) = 0.97; % CI: 0.96-0.98) or highly educated (OR = 1.12; % CI: 1.01-1.24) than non-users among Dutch populations. Nunes et al. discovered that age is a significant predictor of acceptance of information and communication technologies [29]. This could be explained by the high e-health literacy level among younger, more educated adults who are technology-savvy.

There was no statistically significant relationship between users and non-users regarding sex, as similar results were reported among a large sample of the Dutch population (OR = 1.25; 95% CI: 0.94-1.66) [30]. In contrast to the current results, Xie et al. revealed that females were significantly more likely to use health apps than men, particularly with health and medical reminder apps [31]. This is because there are gender differences in attitudes toward healthy living.

The study clearly showed that none of the health-based mobile app users had any form of tobacco consumption. This finding suggests a potential positive impact of health apps on discouraging or preventing tobacco use among university students.

Non-users had a higher prevalence of alcohol consumption compared to health app users. This indicates that individuals who do not use health apps are more likely to engage in alcohol consumption, highlighting a potential association between health app usage and healthier lifestyle choices.

Similar to tobacco and alcohol consumption, the prevalence of unhealthy diets was higher among non-users. This suggests that health app users are more likely to adopt healthier dietary habits compared to their non-user counterparts. Robinson et al. have shown that health-based apps have raised the awareness of users about what they are eating and that they are easy to use [32]. Interestingly, physical inactivity remained similar in both groups. While health apps did not seem to influence physical activity levels, it is essential to explore further why this aspect did not differ between the two groups, which also reflected the same proportion seen in obesity and hypertension status among the groups, which were not significantly affecting the outcome.

Given the absence of tobacco consumption and lesser alcohol consumption among health app users, it is advisable to implement targeted campaigns and interventions promoting health apps as effective tools for cessation. University health services can collaborate with app developers to create tailored programs that address the specific needs and challenges of students quitting tobacco. Health apps should consider incorporating nutrition education and guidance features to help users make healthier dietary choices. Although the study found no significant difference in physical inactivity between the two groups, efforts should be made to explore ways health apps can effectively encourage and track physical activity, which will eventually lead to better body mass index (BMI) status. 

The limitations of the study include that the findings may not be fully generalizable beyond the specific university population studied. The characteristics of students at this particular university may differ significantly from those at other universities or populations. The use of self-reported data through the WHO STEPS questionnaire may introduce response bias. Participants may provide socially desirable responses or inaccuracies regarding their lifestyle practices, leading to an overestimation or underestimation of certain behaviors. The long-term effects of the mobile-based intervention may not be adequately assessed within the study timeframe, and sustained behavior change might not be accurately captured.

## Conclusions

Despite the potential benefits of health-based mobile apps, they were used by only about one-third of the study participants. The use of the apps was significantly higher among younger participants who had no addiction practices and followed healthy diets. The results highlight the potential of health-based mobile apps as effective tools for promoting positive health behaviors among university students. To optimize the role of health-based mobile apps in delivering effective healthcare services, some useful ways, like using social media and healthcare providers as marketing tools for the health-based mobile apps, developing the apps to meet the public's needs and expectations, and improving the apps' usability even with people who have little knowledge about mobile technology, are to be made.
